# Arginine vasotocin is a player in the multifactorial control of spermatogenesis in zebrafish

**DOI:** 10.3389/fendo.2025.1672823

**Published:** 2025-11-03

**Authors:** Maya Zanardini, Nicolas Parker, Yifei Ma, Hamid R. Habibi

**Affiliations:** ^1^ Department of Biological Sciences, University of Calgary, Calgary, AB, Canada; ^2^ Cumming School of Medicine, University of Calgary, Calgary, AB, Canada

**Keywords:** vasotocin (AVT), gonadotropin, follicle-stimulating hormone (FSH), human chorionic gonadotropin (hCG), luteinizing hormone (LH), spermatogenesis, zebrafish

## Abstract

This study investigated the role of vasotocin (AVT) in the multifaceted regulation of spermatogenesis in zebrafish, with a focus on its interaction with gonadotropin hormones. Using an *ex vivo* cultured testis, we explored the interaction of AVT with gonadotropins, LH and FSH, to regulate different stages of germ cell development. In this study, we used recombinant zebrafish FSH and hCG to stimulate the LH-induced response due to the limited availability of zebrafish LH, as it is known to exert similar effects on testicular function. Treatment with AVT enhanced FSH-induced proliferation of early undifferentiated spermatogonia (Aund) germ cells, as well as promoting the proliferation of later-stage type B germ cells when combined with LH/hCG. Additionally, AVT significantly decreased FSH-induced 11-ketotestosterone (11-KT) production and Leydig-derived factors, including *cyp17a1* and *insl3*, without significantly affecting LH/hCG-induced androgen production. Based on these findings, we hypothesize that in the presence of FSH, AVT drives early germ cell proliferation and differentiation while simultaneously inhibiting premature progression through spermiogenesis. This stage-specific modulation of gonadotropin signaling by AVT underscores its dual role in fine-tuning testicular function and germ cell maturation in male zebrafish. Overall, our findings suggest that AVT contributes to the complex multifactorial network regulating spermatogenesis in zebrafish.

## Graphical Abstract

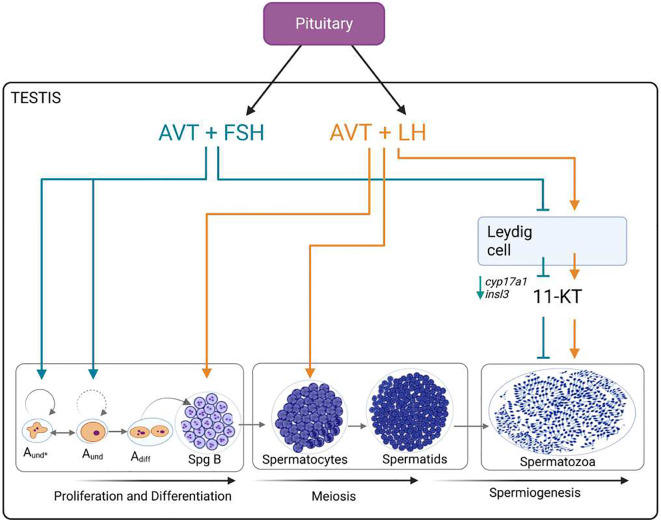

## Highlights

- Vasotocin enhances FSH-induced proliferation of early undifferentiated spermatogonia (Aund) and, when combined with LH/hCG, promotes proliferation at more advanced germ cell stages (SpgB).
- Vasotocin, in the presence of gonadotropins, impairs steroidogenic enzyme transcript levels (*cyp17a1*) consistent with changes in 11-ketotestosterone production.
- Hypothesis: AVT contributes to the multifactorial regulation of spermatogenesis in a stage-specific manner. In the presence of FSH and LH, AVT promoted early germ cell proliferation and differentiation while suppressing premature spermiogenesis.

## 1 Introduction

The hypothalamic-pituitary-gonadal (HPG) axis controls testicular function by regulating the synthesis and release of the gonadotropins follicle-stimulating hormone (FSH) and luteinizing hormone (LH) through neurohormonal signals. Gonadotropins play a crucial role in modulating steroidogenesis and spermatogenesis by acting directly on the testes. In mammals, FSH and LH have distinct functions: FSH promotes germ cell development by acting primarily on Sertoli cells through FSH receptors (FSHR), while LH stimulates steroidogenesis by binding to LH receptors (LHCGR) on Leydig cells. The activity of gonadotropins in fish is less clearly differentiated, with evidence demonstrating overlapping receptor specificity (1, 2). Generally, FSH predominates in early spermatogenesis by promoting spermatogonia proliferation and differentiation, whereas LH is more involved in the later stages, regulating spermiogenesis and sperm release (3). In the African catfish (*Clarias gariepinus*), both FSH and LH receptor transcripts are expressed in Leydig cells, whereas Sertoli cells express only FSHR (4). This pattern is observed in other fish species, including zebrafish (5–9). FSH regulates a number of somatic cell-derived factors crucial for germ cell development, acting largely independent of androgen signaling (10–13). In addition, both FSH and LH interact with gonadal-produced neuropeptides, including gonadotropin-inhibitory hormone (GnIH) and gonadotropin-releasing hormone (GnRH), indicating complex multifactorial regulation of spermatogenesis (14–17). In zebrafish, GnIH inhibits gonadotropin-induced spermatogenesis by suppressing FSH- and hCG-induced androgen production (18, 19). Similarly, it was shown that both GnRH isoforms (GnRH2 and GnRH3) inhibited FSH-induced spermatogenesis, although their effect on hCG-induced spermatogenesis was less pronounced (17). There is evidence that the neurohypophysial hormone arginine vasotocin (AVT) may also be a player in the regulation of spermatogenesis. In this context, multiple studies have demonstrated the expression of AVT in the testes of several fish species, including zebrafish (20–26). In addition, vasotocin receptors were also found to be expressed in the testes of different species, including catfish and zebrafish (24, 26). *In vitro* experiments reported that AVT stimulated 11-ketotestosterone and testosterone in the cichlid fish *Cichlasoma dimerus* and the rainbow trout *Oncorhynchus mykiss*, respectively (23, 27). Consistent with these findings, we recently demonstrated that treatment of cultured adult zebrafish testes with zebrafish AVT increased 11-KT levels and spermatozoa production while reducing type B spermatogonia proliferation (26). These findings indicate that AVT acts directly on spermatogenesis in an androgen-dependent manner. However, only a limited number of studies have investigated the interaction between vasotocin and gonadotropins in the gonads, and those available primarily focus on the regulation of pituitary gonadotropin production. Ramallo et al. (23) demonstrated that vasotocin modulates pituitary secretion of LH and FSH *in vitro* in a dose-dependent and hormone-specific manner; LH exhibited a triphasic response and FSH levels increased progressively with rising vasotocin concentrations. Also, in female Asian stinging catfish (*Heteropneustes fossilis*), *in vitro* treatment of ovarian follicles with hCG stimulated vasotocin production; additionally, hCG injection enhanced vasotocin levels in the brain, ovary, and plasma (28). These results demonstrate the involvement of vasotocin in the control of reproduction. However, the downstream physiological effects of AVT-gonadotropin interactions in the male gonads, testicular function and spermatogenesis, remain largely unexplored.

Building on our previous work, we investigated how arginine vasotocin (AVT) affects spermatogenesis and androgen production in the presence of gonadotropins. Using an *ex vivo* zebrafish testis culture, we examined the interactions of LH/hCG and FSH with AVT by assessing germ cell development, proliferation of early spermatogonia, androgen synthesis, and transcript levels.

## 2 Material and methods

### 2.1 Hormones and chemicals

The zebrafish-specific (Arg^8^)-Vasotocin peptide was purchased from ChinaPeptides Co.,Ltd (Shanghai, China) (CYIQNCPRG with C1-C6 bridge). This hormone was reconstituted with 1x PBS and then diluted with filter-sterilized Leibovitz’s L-15 Medium (Thermo Fisher Scientific, Gibco^®^, Canada) to reach a final working concentration of 10 nM.

Recombinant zebrafish follicle-stimulating hormone (rzfFSH) was purchased from U-Protein Express B.V. (Utrecht, The Netherlands). The hormone was reconstituted with 1x PBS without preservative, aliquoted, and stored at -20 °C until use. Based on previous studies in zebrafish (12, 19), we initially tested a concentration of FSH of 100 ng/ml without seeing any effect on spermatogenesis or gene expression, possibly due to a different batch of the FSH hormone being produced. We therefore decided to increase the concentration to 200 ng/ml. Hence, for *ex vivo* experiments, the hormone was diluted with L-15 medium to reach the final concentration of 200 ng/ml.

Human chorionic gonadotropin (hCG) was purchased from Sigma-Aldrich (Oakville, ON, Canada), reconstituted with ultrapure distilled water, and stored at -20 °C until use. The hormone was diluted with L-15 medium to reach the final concentration of 10 IU/ml for tissue culture.

### 2.2 Experimental animals

Adult zebrafish (*Danio rerio*, TL strain) were bred and raised in the Department of Biological Sciences aquatic facility at the University of Calgary (Alberta, Canada). Fish were maintained in a partially recirculating system with water maintained at 28 °C, pH 7.6, and conductivity 750 μS, and were fed twice daily with the adult commercial food Zeigler^®^ (Pentair Aquatic Habitats). Male zebrafish between 6 and 10 months old were used for this study. Fishes were fasted for 18 hours before all experiments to standardize physiological conditions and reduce contamination from intestinal contents during testis dissection and *ex vivo* culture preparation. All procedures described here follow the guidelines of the Canadian Council on Animal Care (CCAC) and have been approved by the University of Calgary Animal Care Committee (protocol# AC24-0042).

### 2.3 *Ex vivo* testis culture

Testes were dissected from zebrafish and incubated with L-15 medium using an *ex vivo* system, as described by Leal et al. (29). Testes were placed on a nitrocellulose membrane positioned on 2% agarose blocks surrounded by the culture medium. For each treatment, testes were incubated with L-15 alone to assess basal spermatogenesis or with L-15 supplemented with rzfFSH, LH/hCG, or AVT, alone and in combination (n=6-8). Culture plates were kept for 7 days at 28 °C in a humidified controlled environment to evaluate the effect of AVT on gonadotropin-induced spermatogenesis and androgen production, and 48 hours for transcript abundance measurement. A complete medium change was performed on day three during the 7-day incubation period.

### 2.4 Histology and morphometric analysis

To analyze testicular morphology and quantify the distribution of different stages of germ cells after 7 days of culture, testes were collected and fixed overnight in 1.6% paraformaldehyde, 2.5% glutaraldehyde, and PBS 1× (pH: 7.2), as described previously (19, 26). Tissues were washed in PBS 1× and dehydrated in ethanol series the following day. Testes explants were then embedded in Technovit 7100 (Heraeus Kulzer, Wehrheim, Germany), and histology blocks were sectioned using a Reichert-Jung 2040 Autocut Rotary Microtome. Slices of 3 μm thickness were cut, stained with 0.1% Toluidine Blue, and mounted using Kristalon Mounting Medium (Sigma-Aldrich, Burlington, MA, USA). Bright-field images of five images per sample were taken, and an area of 20000 µm^2^ was used to quantify the germ cells. In histology sections, germ cells were counted and classified into developmental stages: spermatogonia type Aund*+Aund (where the asterisk indicates stem cell), spermatogonia type Adiff, spermatogonia type B, spermatocytes, and spermatozoa. Cell classification was based on morphological criteria according to Schulz et al. (3), enabling identification of testicular germ cell types by distinct characteristics observed under microscopy.

### 2.5 BrdU proliferation assay

To evaluate the spermatogonia germ cells proliferation rate, we carried out immunohistochemistry using BrdU (Bromodeoxyuridine/5-bromo-2’-deoxyuridine), a thymidine analog that incorporates into replicating DNA (S-phase of cell cycle). A pulse of 100 μg/mL BrdU was given to testes incubated in the *ex vivo* system 6 hours before the collection of the 7-day culture period. The protocol followed was described by Zanardini et al. (26). The BrdU labeling index was calculated as the percentage of BrdU-positive cells relative to the total number of spermatogonia, including type A undifferentiated (Aund), type A differentiated (Adiff), and type B spermatogonia. Bright-field images were captured from five randomly selected areas per sample, each covering 20,000 µm² for germ cell quantification, consistent with the approach used in histological analysis.

### 2.6 RNA extraction, cDNA synthesis and qPCR

Molecular analyses were conducted using quantitative PCR (qPCR) following a protocol adapted from previous studies (26). After a 48-hour *ex vivo* incubation, testes were rapidly frozen for RNA preservation. Total RNA was extracted using TRIzol reagent (Invitrogen, Canada), and RNA quality and quantity were assessed via Nanodrop 2000 spectrophotometry (Thermo Scientific, Waltham, MA, USA). To eliminate genomic DNA contamination, samples were treated with RNase-free DNase I (Thermo Fisher Scientific) prior to cDNA synthesis using the iScript™ cDNA Synthesis Kit (Bio-Rad, Mississauga, Ontario, Canada). qPCR reactions utilized SsoFast Eva Green Supermix (Bio-Rad) and were performed in duplicate. Gene expression levels were normalized using *eef1a1l1* (*elongation factor 1 alpha 1, like 1*) as the reference gene, with relative quantification calculated through the 2^−ΔΔCT^ method. Primer sequences were selected based on previously published work (26). In the table, the primer labeled as ‘*lhr*’ corresponds to the luteinizing hormone receptor gene, whose current official gene symbol is *lhcgr*.

### 2.7 Quantification of androgen release by ELISA

After a 7-day incubation period, the culture medium was collected to assess androgen release. The concentration of 11-ketotestosterone (11-KT) secreted by testes under different treatment conditions was quantified using an Enzyme-Linked Immunosorbent Assay (ELISA) kit (Cayman Chemicals, Ann Arbor, MI, USA). Measurements of the enzymatic reaction product were performed with a SpectraMax i3 microplate reader (Molecular Devices, San Jose, CA, USA) set to an absorbance wavelength of 412 nm. Prior to culture, testes were weighed individually, and 11-KT levels were normalized to tissue mass and expressed as picograms per milligram.

### 2.8 Statistical analysis

All raw data from each experiment and endpoint were first assessed for normal distribution using the Shapiro-Wilk test, and outliers were evaluated and removed using the ROUT method (30). Data from histology were analyzed using unpaired T-tests. Data from 11-KT measurement, transcript level, and immunohistochemistry were analyzed with one-way ANOVA followed by Tukey’s multiple comparison test. Differences were considered significant when P<0.05. All statistical tests were performed using GraphPad Prism 8.0 software (GraphPad Software Inc., La Jolla, CA, USA).

## 3 Results

### 3.1 Effect of AVT on gonadotropin-induced spermatogenesis

**Figure 1:**
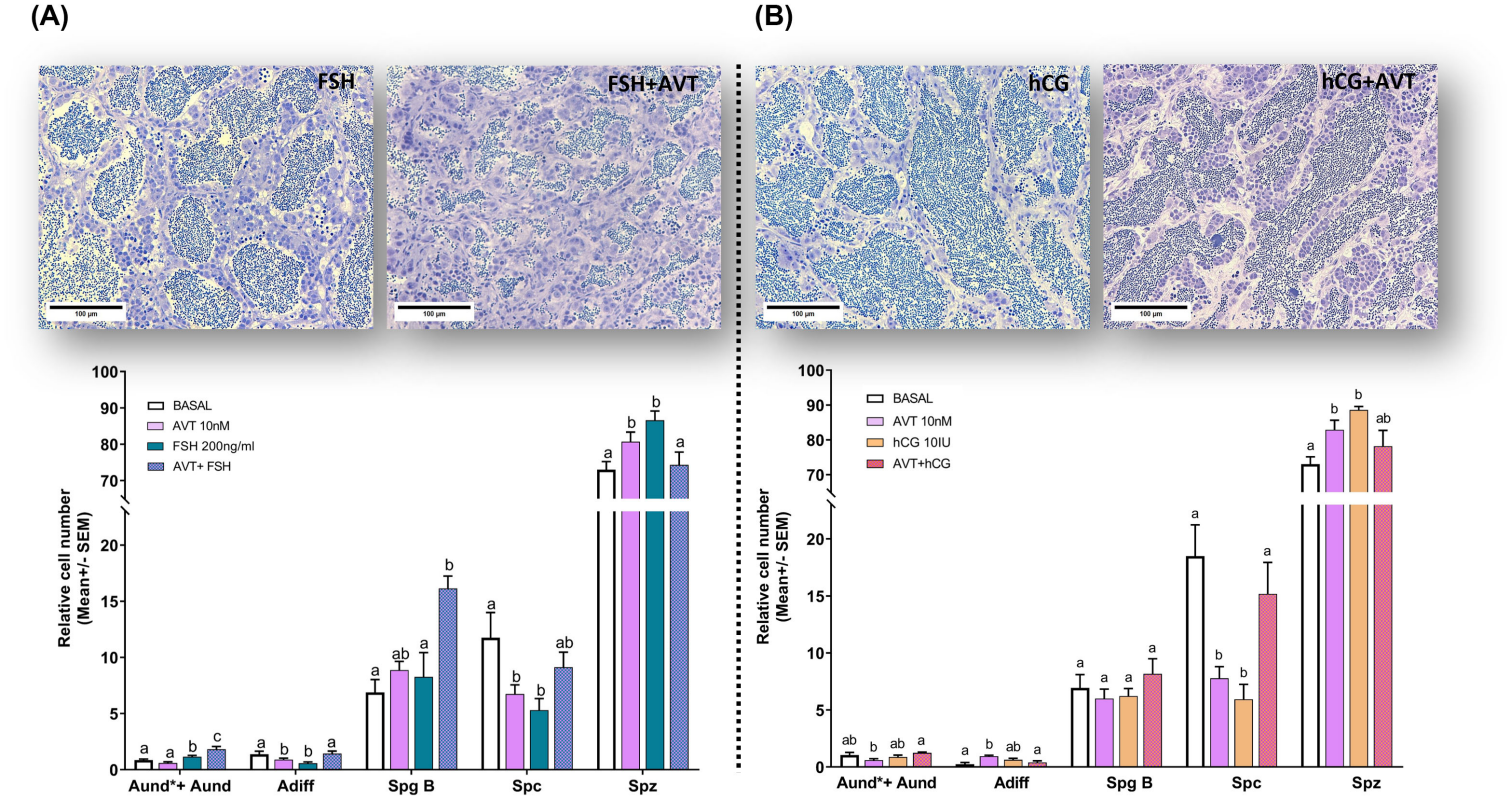
Effect of vasotocin on **(A)** FSH- and **(B)** LH/hCG-induced spermatogenesis. Representative histological sections (upper panels) are shown for testes treated with FSH (200 ng/ml) or LH/hCG (10 IU), alone or in combination with AVT (10 nM), for 7 days in ex vivo culture. Quantitative morphometric data (lower panels) summarize relative cell numbers (mean ± SEM; normalized to control) for undifferentiated spermatogonia (Aund*+Aund), differentiating spermatogonia (Adiff), type B spermatogonia (Spg B), spermatocytes (Spc), and spermatozoa (Spz), following treatments with L-15 medium (Control), AVT (10 nM), FSH (200 ng/ml), LH/hCG (10 IU), or the indicated combinations. Different letters denote significant differences among treatments within each cell type (p < 0.05; one-way ANOVA, Tukey’s test). Scale bars: 100 μm.

In the present study, we investigated the effect of vasotocin (10 nM) on LH- and FSH-induced spermatogenesis using morphometric analysis. Our previous work identified 10 nM AVT as the optimal concentration for directly stimulating spermatogenesis over 7 days of treatment (26). Representative histological sections are shown for testes treated with FSH or LH/hCG, alone or in combination with AVT, primarily to illustrate the relative abundance of spermatozoa cysts, whereas the quantitative analysis includes all treatment groups (Control, AVT, FSH, LH/hCG, and combinations) (**Figure 1**). Treatment with FSH (200 ng/ml) alone significantly increased the number of undifferentiated spermatogonia (Aund*+Aund) while reducing differentiated spermatogonia (Adiff) (**Figure 1A**). By contrast, co-treatment with AVT and FSH significantly increased the number of Aund*+Aund, Adiff, and type B spermatogonia (SpgB) compared with either treatment alone (**Figure 1A**). LH/hCG (10 IU), with or without AVT, had no effect on early spermatogonial stages (Aund*+Aund, Adiff, SpgB) (**Figure 1B**).

Treatment with AVT or FSH alone reduced spermatocyte numbers, whereas their combined administration produced a modest but non-significant increase. In contrast, either treatment alone increased spermatozoa proportion, while co-treatment reversed this effect, resulting in a reduction (**Figure 1A**). Treatment with either LH/hCG or AVT alone significantly reduced the number of spermatocytes and increased the number of spermatozoa. In contrast, combined treatment with AVT and LH/hCG increased the number of spermatocytes but led to a non-significant reduction in spermatozoa compared to single treatments (**Figure 1B**).

### 3.2 Effect of AVT on early germ cell proliferation

**Figure 2:**
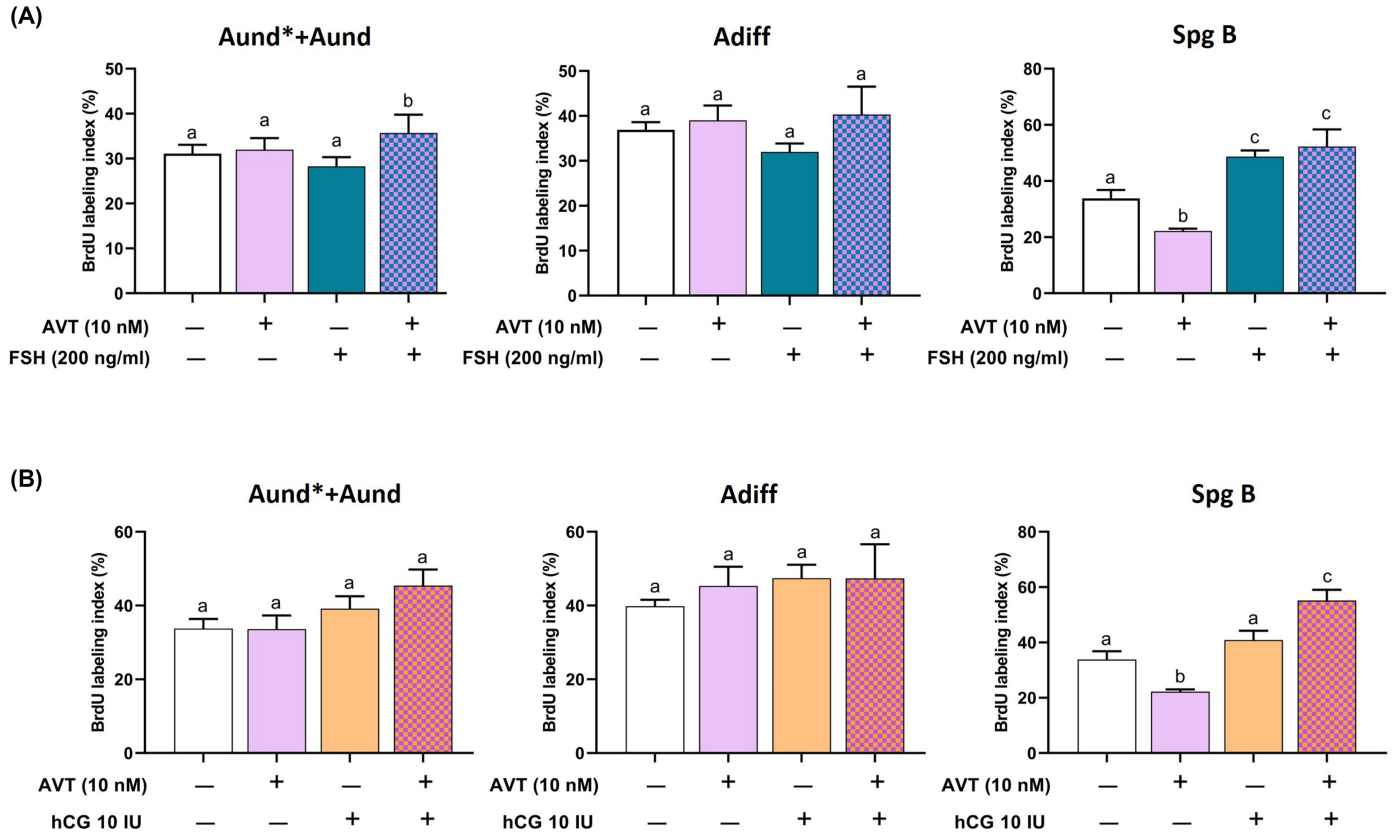
Proliferation activity of pre-meiotic germ cells in zebrafish testis explants treated with AVT in combination with FSH or LH/hCG. Testis were treated for 7 days ex vivo with AVT (10 nM), FSH (200 ng/ml), or LH/hCG (10 IU), alone or in combination, and analyzed for BrdU incorporation to assess spermatogonial proliferation. BrdU labeling index is presented as a percentage of BrdU-positive cells vs the total number of cells, per each cell type analyzed. **(A)** BrdU labeling index (%) for undifferentiated spermatogonia (Aund* + Aund), differentiating spermatogonia (Adiff), and type B spermatogonia (Spg B) following treatment with AVT and/or FSH. **(B)** Same cell populations analyzed following treatment with AVT and/or LH/hCG. Different letters indicate statistically significant differences among treatment groups within each cell type (p < 0.05, Tukey’s multiple comparison test).

To assess spermatogonia proliferation, we measured BrdU mitotic index following treatment with FSH or LH/hCG alone, and in combination with AVT (**Figure 2**). FSH alone did not alter the mitotic index for Aund*+Aund or Adiff but significantly increased proliferation of SpgB (**Figure 2A**). Co-treatment with AVT and FSH further enhanced the proliferation of Aund*+Aund compared to FSH alone while promoting SpgB proliferation at a similar level as FSH treatment alone (**Figure 2A**). LH/hCG alone had no effect on the mitotic index of diploid spermatogonia (**Figure 2B**). In contrast, co-treatment with AVT and LH/hCG significantly increased SpgB proliferation compared to either treatment alone (**Figure 2B**).

### 3.3 Effect of AVT on gonadotropin-induced androgen production

**Figure 3:**
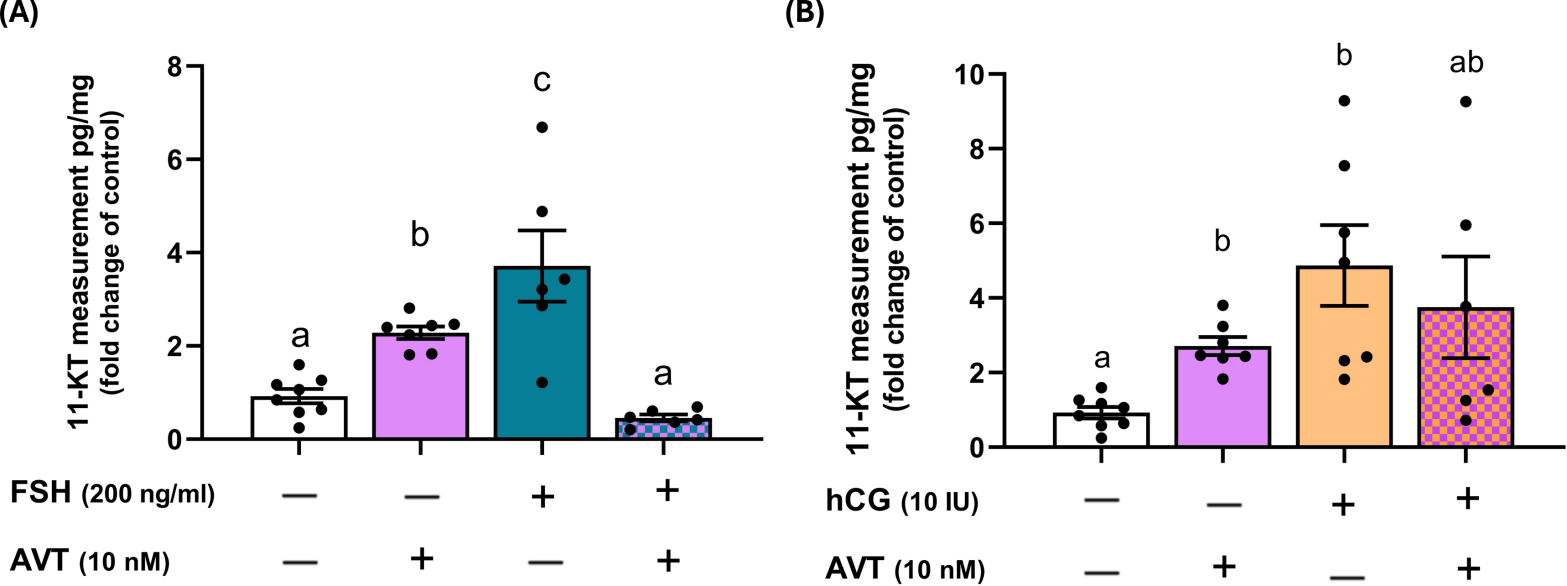
Effects of AVT on gonadotropin-induced 11-ketotestosterone (11-KT) production in zebrafish testis explants. Testes were cultured and treated with AVT (10 nM), either alone or in combination with **(A)** FSH (200 ng/ml) or **(B)** LH/hCG (10 IU). 11-KT concentrations were measured in the culture medium and expressed as fold changes relative to their respective contralateral control testes (pg/mg of tissue). Different letters denote statistically significant differences among treatment groups (p < 0.05, ANOVA followed by Tukey’s multiple comparison test).

Androgens are critical for the final stages of germ cell development and spermatogenesis. To evaluate androgen release, we measured 11-ketotestosterone (11-KT), a non-aromatizable androgen, following treatment of zebrafish testes with AVT, FSH, and LH/hCG, individually and in combination (**Figure 3**). Both AVT and FSH alone significantly increased 11-KT secretion; however, combined treatment with FSH and AVT markedly reduced 11-KT levels compared to either treatment alone (**Figure 3A**). Similarly, LH/hCG alone stimulated 11-KT production, whereas the addition of AVT did not modify the LH/hCG-induced response (**Figure 3B**).

### 3.4 Effect of AVT, FSH, and LH/hCG on selected transcript levels

**Figure 4:**
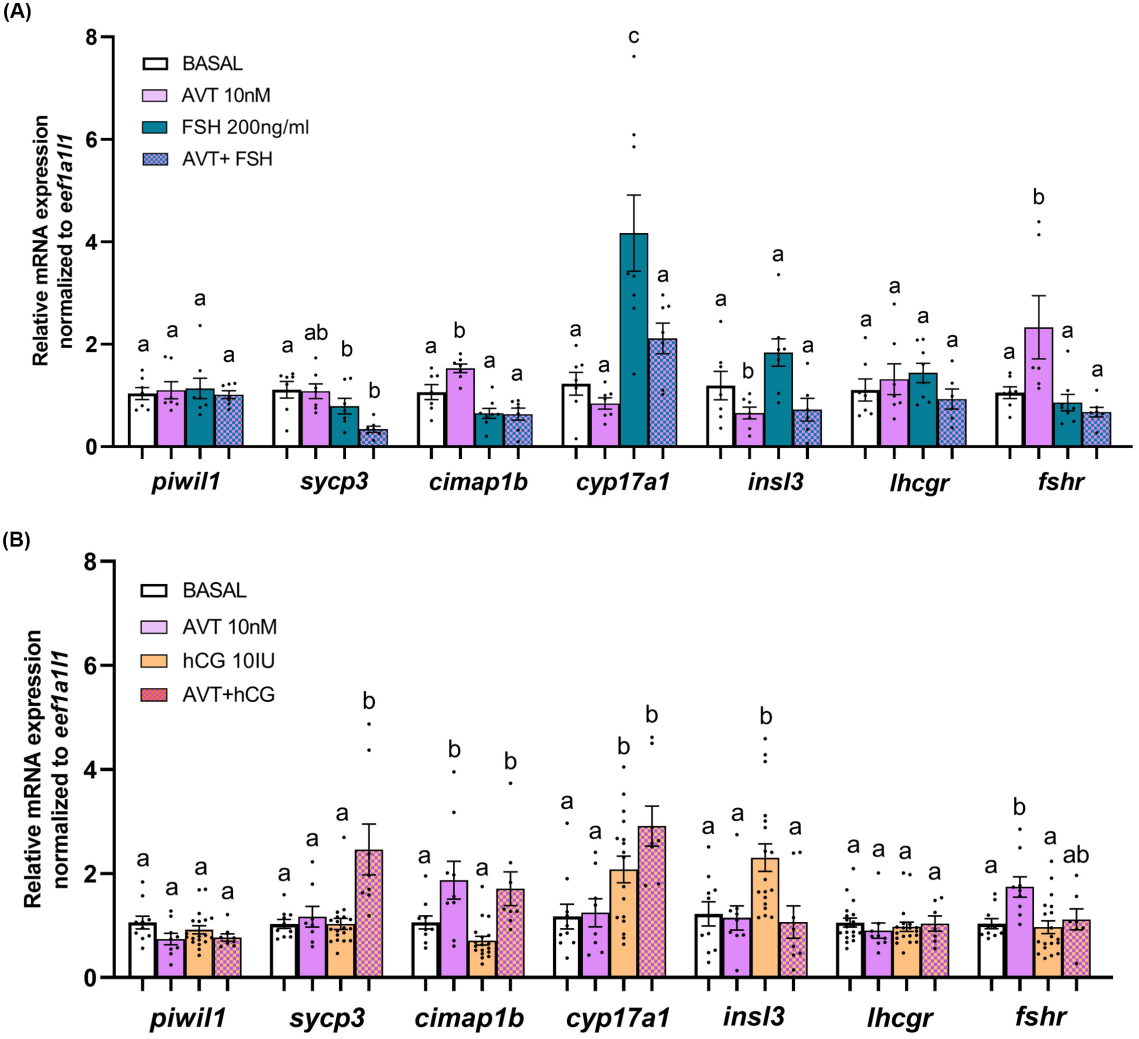
Relative transcript abundance of selected transcripts in zebrafish testes after 48-hour ex vivo treatment with AVT, FSH, LH/hCG, or their combinations. Testes were treated ex vivo for 48 hours with **(A)** AVT (10 nM) and FSH (200 ng/ml) alone or in combination, or **(B)** AVT (10 nM), LH/hCG (10 IU/ml), or their combination. Expression levels of germ cell markers (*piwil1*, *sycp3*, *cimap1b*), steroidogenic enzyme *cyp17a1*, Leydig-derived factor *insl3* and the gonadotropin receptors (*lhcgr*, *fshr*) were quantified by RT-qPCR. Basal expression represents non-treated testes. All treatments are shown as fold change relative to the respective contralateral control. Data are presented as mean ± SEM (n = 6-10). Statistical differences between each treatment and its control were assessed using Student’s t-test. Different letters denote statistically significant differences among treatment groups determined by one-way ANOVA followed by Tukey’s *post hoc* test (p < 0.05).

To further examine the effects of AVT on germ cells, we analyzed transcript levels of germ cell-specific markers: *piwil1* (type A undifferentiated and differentiated spermatogonia), *sycp3* (spermatocytes), and *cimap1b* (spermatids and spermatozoa). AVT treatment did not alter the FSH-induced transcript levels of *piwil1*, *sycp3*, or *cimap1b* (**Figure 4A**). In contrast, co-treatment with AVT and LH/hCG increased the expression of *sycp3* and *cimap1b*, consistent with enhanced spermatocytes and haploid cells, partially aligning with histological observations (**Figure 4B**).

We also measured Leydig-derived transcripts, including *cyp17a1* (steroidogenic enzyme), *insl3* (promotes type Aund spermatogonia differentiation), and the gonadotropin receptors *lhcgr* and *fshr*. In the presence of FSH, co-treatment with AVT significantly reduced *cyp17a1* and *insl3* expression compared to FSH alone (**Figure 4A**). Gonadotropin receptor transcript levels were not affected by FSH treatment; however, AVT in combination with FSH suppressed AVT-induced *fshr* expression. LH/hCG alone or in combination with AVT upregulated *cyp17a1* transcript, whereas AVT inhibited the LH/hCG-induced *insl3* transcript level (**Figure 4B**). Transcript levels of *lhcgr* and *fshr* remained unaffected following co-treatment with LH/hCG and AVT.

## 4 Discussion

Limited data exist on the role of arginine vasotocin (AVT) in the multifactorial regulation of fish gonadal function. The present study provides the first evidence that AVT interacts directly with gonadotropins to regulate testicular function in teleost. By investigating its actions in *ex vivo* cultured zebrafish testes, we show that AVT acts as a stage-specific modulator of gonadotropin signaling, influencing both spermatogonial proliferation and steroidogenesis in a hormone-dependent manner.

Our findings demonstrate that in the presence of FSH, AVT stimulates the proliferation of undifferentiated type A spermatogonia. This was reflected in increased numbers of Aund*+Aund and enhanced mitotic activity, indicating that AVT amplifies the proliferative effect of FSH at the earliest stage of spermatogenesis. Interestingly, although the accumulation of type A differentiated spermatogonia also increased, the mitotic index did not rise accordingly, suggesting that this effect is cumulative rather than mitogenic. AVT also enhanced the FSH-induced expansion of type B spermatogonia, thereby potentiating FSH activity in both self-renewal and differentiation processes (12, 31). These findings highlight a stage-specific co-regulatory role for AVT in promoting the development of the early germ cell pool under FSH stimulation. Consistent with earlier studies (19), our results indicate that LH/hCG alone is insufficient to trigger proliferation of undifferentiated spermatogonia, suggesting that LH-like signaling plays only a limited role at the onset of spermatogenesis. However, when combined with AVT, LH/hCG promoted the proliferation of type B spermatogonia, demonstrating that AVT can potentiate LH activity at more advanced stages of germ cell development. The distinction between FSH-regulated early spermatogenesis and LH-mediated effects on advanced stages, such as spermiogenesis, is well documented in other teleosts (32, 3). In this context, our results identify AVT as a potentiator of gonadotropin function, amplifying FSH signaling to expand the pool of undifferentiated spermatogonia while simultaneously enhancing LH-like activity to promote proliferation at later germ cell stages, such as SpgB.

In addition to its effects on germ cell proliferation, AVT also displayed hormone-specific modulation of steroidogenesis. As expected, both FSH and LH independently stimulated 11-KT production and upregulated key steroidogenic genes, including *cyp17a1* and *insl3*. However, when combined with AVT, the FSH-induced increase in 11-KT release and steroidogenic gene expression was significantly attenuated, while the LH-induced response remained unaffected. This pattern suggest that AVT selectively constrains FSH-driven steroidogenesis, an effect consistent with rodent studies where vasopressin modulated testosterone output in a time- and hormone-dependent manner (33). Such a mechanism would allow AVT to promote early germ cell expansion under FSH while limiting premature androgen-driven completion of spermatogenesis.

The gene expression results provide further insight into these interactions. Neither FSH nor LH strongly altered germ cell marker transcripts, except for a modest FSH-induced repression of *sycp3*. Notably, the addition of AVT to LH/hCG, but not to FSH, upregulated *sycp3* and *cimapb1*, markers of meiotic and post-meiotic germ cells, respectively. These results, consistent with the histological data, indicate that AVT enhances LH-like effects at the level of meiotic progression. Furthermore, the combined action of AVT and FSH triggered a specific downregulation of the *fshr* transcript, without impacting *lhcgr*. This selective receptor regulation may reflect a negative feedback mechanism that prevents overstimulation when FSH and AVT act together. Similar gonadotropin receptor downregulation under sustained stimulation has been documented in zebrafish (6) and in mammals (34, 35), suggesting a conserved self-limiting response across vertebrates. In our study, the presence of AVT appears to potentiate FSH signaling to a threshold that activates this feedback control. The absence of a comparable effect on LH receptor expression highlights the specificity of AVT’s interaction with FSH pathways, pointing to a finely tuned regulatory mechanism that balances proliferation and steroidogenic activity during spermatogenesis.

Our earlier work demonstrated the expression of *avt* and its five receptors (*avpr1aa*, *avpr1ab*, *avpr2aa*, *avpr2ab*, *avpr2l*) in zebrafish testes, pointing to a direct role of AVT in testicular regulation (26, 36). The present findings extend this evidence by identifying AVT as a local paracrine/autocrine factor that fine-tunes gonadotropin signaling in a stage-specific manner. By enhancing FSH-driven expansion of undifferentiated spermatogonia, reinforcing LH-mediated proliferation of type B spermatogonia, and selectively dampening FSH-induced steroidogenesis, AVT coordinates the balance between germ cell proliferation and differentiation. We propose that this dual role enables the zebrafish testis to expand the pre-meiotic pool while restraining premature androgen-dependent spermiogenesis, helping to maintain germ cell synchrony and ensure reproductive success.

Based on these results, we hypothesize that AVT plays a stage-specific modulatory role: it is crucial for enhancing FSH activity during the earliest phases of spermatogonial proliferation and for potentiating LH activity at mid-spermatogonial stages, but its influence diminishes once germ cells progress toward spermiogenesis. At this point, LH alone appears sufficient to sustain androgen production and haploid cell formation, as indicated by the unchanged 11-KT levels and haploid cell responses to LH/hCG regardless of AVT presence. Supporting this view, reduced *avt* expression levels have also been observed in testes of zebrafish immediately prior to spawning events (unpublished; 36).

Collectively, our findings underscore the role of nonapeptides in the multifactorial control of male reproduction and encourage broader investigation into their evolutionary and physiological significance.

While this work provides novel insight into the role of AVT in modulating gonadotropin signaling, some limitations should be noted. First, the use of an *ex vivo* testis culture model, although powerful for dissecting direct effects, cannot fully capture the complexity of *in vivo* endocrine, paracrine, and environmental interactions that modulate and affect spermatogenesis. Second, the gene expression analyses focused on a restricted set of markers involved in spermatogenesis and steroidogenesis; broader transcriptomic or proteomic approaches would be valuable to reveal additional downstream targets of AVT–gonadotropin interactions. Finally, the conclusions are based on zebrafish, and although this species is as an established teleost model, the mechanisms observed may display species-specific features that require further investigation and validation in other vertebrates. Addressing these limitations in future studies will be essential to confirm our findings across different conditions and provide a more comprehensive understanding of AVT’s role in regulating testicular function.

## Data Availability

The original contributions presented in the study are included in the article/supplementary material. Further inquiries can be directed to the corresponding author.
